# Protective effects of cilostazol via the HNF1α/FXR signalling pathway and anti-apoptotic mechanisms in a rat model of estrogen-induced intrahepatic cholestasis

**DOI:** 10.1038/s41598-024-72729-w

**Published:** 2024-10-01

**Authors:** Marwa Hassan, Maha B. Salem, Olfat A. Hammam, Mohamed ElZallat

**Affiliations:** 1 Immunology Department, Theodor Bilharz Research Institute, Warrak El-Hadar, P.O. box 30, Imbaba, 12411 Giza Egypt; 2 Pharmacology Department, Theodor Bilharz Research Institute, El-Nile St., Warrak El-Hadar, P.O. box 30, Imbaba, 12411 Giza Egypt; 3 Pathology Department, Theodor Bilharz Research Institute, Warrak El-Hadar, P.O. box 30, Imbaba, 12411 Giza Egypt

**Keywords:** Intrahepatic cholestasis, Cilostazol, HNF1α/FXR pathway, CYP7A1, Apoptosis, Biochemistry, Immunology, Diseases

## Abstract

Currently, there is a lack of targeted medications for estrogen-induced intrahepatic cholestasis (EIC) and the primary objective in managing this condition is to safeguard liver function. Consequently, this study was conducted to examine the pharmacological efficacy of cilostazol (CTZ) in the management of EIC and explore its underlying mechanisms through the use of an animal model. Thirty female Sprague-Dawley rats were divided into five groups of six animals each: Normal group, 17-ethinylestradiol (EE)-induced intrahepatic cholestasis group, EE + ursodeoxycholic acid (UDCA)-treated group, EE + CTZ (5 mg/kg)-treated group, and EE + CTZ (10 mg/kg)-treated group. It was found that the therapeutic efficacy of UDCA and low dosage of CTZ (5 mg/kg) was comparable. Nevertheless, when CTZ was administered at a dose of 10 mg/kg, it resulted in the normalization of all liver function parameters, oxidative stress, and pro-inflammatory markers, together with improvement in the histopathological derangements and hepatocytic apoptosis. These effects were mediated through the activation of the hepatocyte nuclear factor-1 alpha (HNF1α)/Farnesoid X receptor (FXR) pathway with subsequent down-regulation of the bile acids (BAs) synthesis enzyme; cholesterol 7α-hydroxylase (CYP7A1), and up-regulation of the BAs-metabolizing enzyme; cytochrome P450 (CYP)3A1 and the bile salt export pump; BSEP. Therefore, the administration of CTZ in a dose-dependent manner can protect against EIC through regulating the HNF1α/FXR pathway and anti-apoptotic mechanisms. This implies that CTZ exhibits considerable promise as a therapeutic agent for the treatment of cholestatic liver disorders.

**Supplementary Information:**

The online version contains supplementary material available at 10.1038/s41598-024-72729-w.

## Introduction

Cholestasis is a syndrome characterized by disturbed bile secretion and flow^1^. It may result from hepatocellular functional impairment in the secretion of bile and/or from an obstruction at any level of the bile excretory pathway leading to retention of the bile constituents, such as bilirubin and bile acids (BAs), in the blood. It is classified into intrahepatic and extrahepatic cholestasis^2^. The extrahepatic cholestasis arises from blockage of bile flow outside the liver whereas the intrahepatic cholestasis is caused by a hepatocellular condition that renders the hepatocyte unable to metabolize and excrete bile^1^. The intrahepatic accumulation of cytotoxic BAs provokes cholestatic liver injury, which manifests as hepatocellular integrity disruption, inflammation, fibrosis, cirrhosis, and elevated risk of cancer development^3,4^.

Estrogens are sex steroid hormones that have critical functions in regulating the female reproductive system, bone density, cholesterol mobilization, electrolyte balance, brain function, cardiovascular system, and central nervous system^5^. However, estrogens and their metabolites can induce cholestasis in pregnant and postmenopausal women who receive oral contraception or hormone replacement therapy, particularly in genetically predisposed individuals^5^.

Estrogen-induced cholestasis (EIC) is a disease with a still unidentified etiology and an incidence ranging from 0.2 to 5.6% that varies greatly among ethnic groups and different geographic regions^6^. It frequently manifests as unexplained generalized pruritus in combination with increased serum BAs and/or transaminases in the late second or third trimesters^7^. Despite the maternal prognosis being favorable, the disease has been linked to an increased risk of adverse perinatal outcomes including meconium-contaminated amniotic fluid, preterm labor, and stillbirth^8^. To date, there is no specific medication for EIC, and the major goal is to preserve the liver and lower the cholic acid level in order to improve clinical pregnancy outcomes^9^.

Although the exact pathogenesis of EIC is elusive, genetic, immunological, nutritional, and environmental factors are believed to be implicated^10^. Estrogens can cause cholestasis by interfering with the synthesis, metabolism, and transport of BA, resulting in downstream disruption of the BAs homeostasis and a reduction in the bile flow. Consequently, the BAs accumulate in the liver inducing oxidative stress and pro-inflammatory cytokines expression, further causing cholestatic liver injury^11^.

One of the most crucial sensors in maintaining the BAs homeostasis is the hepatic nuclear receptor, farnesoid X receptor (FXR) and hepatocyte nuclear factor 1α (HNF1α) which regulates the levels of BAs transporters and synthesizing and metabolizing enzymes^4^. Estrogens and their metabolites can suppress the FXR expression, which in turn induces the BAs synthesis enzymes [cholesterol 7α-hydroxylase (CYP7A1) and sterol 12α-hydroxylase (CYP8B1)], inhibits the detoxifying enzymes [sulfotransferase family 2 A member 1 (SULT2A1), cytochrome P450 (CYP) 2 isoforms, and CYP3A], and decreases the expression of BAs transporters in the liver’s canalicular membranes^12^. As a result, BAs may be retained in the hepatocytes with a change in their composition^13^. Hence, targeting FXR can serve as a prospective therapeutic target for cholestatic liver disorders.

Cilostazol (CTZ) is a phosphodiesterase III inhibitor that increases the intracellular cyclic AMP levels by blocking its hydrolysis, leading to the inhibition of hepatic stellate cell activation^14^. The pharmacological effects of CTZ include vasodilation, suppression of vascular smooth muscle cell growth, inhibition of platelet activation and aggregation and thrombus formation, and alleviation of inflammation^15^. It is commonly used in the treatment of peripheral artery disease in the lower extremities^16^. Recently, it has been demonstrated that CTZ attenuates cholestatic liver injury, ameliorates liver functions, and reduces portal hypertension and hepatic fibrosis^17^. However, the effects and mechanisms underlying CTZ’s pharmacological efficacy in EIC remain unknown. Therefore, the present study was conducted to elucidate the potential mechanisms of CTZ activity in an experimental model of cholestasis generated through the administration of 17α-Ethinylestradiol (EE) which is widely used to investigate the underlying molecular/cellular mechanisms involved in EIC.

## Results

### CTZ treatment alleviated liver damage and oxidative stress markers

The subcutaneous injection of EE caused a significant elevation in the serum levels of alanine aminotransferase (ALT), aspartate aminotransferase (AST), alkaline phosphatase (ALP), total bilirubin, and direct and indirect bilirubin when compared to the normal control group. Whereas, the UDCA treatment reduced the EE-induced rise in ALT, AST, total bilirubin, direct bilirubin, and indirect bilirubin levels by 39.66%, 28.69%, 55.75%, 65.71%, and 48.29%, respectively. Also, administration of CTZ at a dose of 5 mg/kg reduced the increase in serum ALT, AST, ALP, total bilirubin, direct bilirubin, and indirect bilirubin by 42.88%, 28.61%, 42.99%, 44.50%, 58.29%, and 34.19%, respectively. Nonetheless, 10 mg/kg CTZ led to the restoration of liver function markers and bilirubin levels (Table 1).

**Table 1:** Effect of CTZ on serum biochemical makers.

Animal groups		ALT (IU/mL)	AST (IU/mL)	ALP (IU/mL)		Total bilirubin (mg/dL)		Direct bilirubin (mg/dL)			Indirect bilirubin (mg/dL)
Normal		67.30 ± 4.30	134.17 ± 5.79	79.28 ± 1.35		0.52 ± 0.05		0.18 ± 0.03			0.34 ± 0.06
EE		158.11 ± 4.22^a^	250.46 ± 5.71^a^	177.54 ± 6.32^a^		4.09 ± 0.31^a^		1.75 ± 0.24^a^			2.34 ± 0.16^a^
EE + UDCA		95.40 ± 7.95^ab^	178.61 ± 8.32^ab^	93.27 ± 5.60^b^		1.81 ± 0.28^ab^		0.60 ± 0.21^ab^			1.21 ± 0.26^ab^
EE + CTZ (5 mg/kg)		90.31 ± 1.85^ab^	178.80 ± 13.00^ab^	101.22 ± 7.15^ab^		2.27 ± 0.28^ab^		0.73 ± 0.26^ab^			1.54 ± 0.30^ab^
EE + CTZ (10 mg/kg)		78.16 ± 4.04^c^	156.53 ± 11.22^b^	79.98 ± 3.78^b^		0.79 ± 0.16^b^		0.21 ± 0.04^bcd^			0.59 ± 0.16^b^

Values presented are means of 6 rats ± SEM. Statistical analysis was carried out using one-way ANOVA followed by a Tukey’s-*post hoc* test. *ALT* alanine aminotransferase, * AST* aspartate aminotransferase, * ALP* alkaline phosphatase, * EE* 17α-ethinylestradiol, * UDCA* ursodeoxycholic acid, * CTZ* cilostazole. ^a, b, c, d^Significantly different from normal, EE, EE + UDCA, and EE + CTZ (5 mg/kg) groups at *p* < 0.05, respectively.

**Fig. 1:**
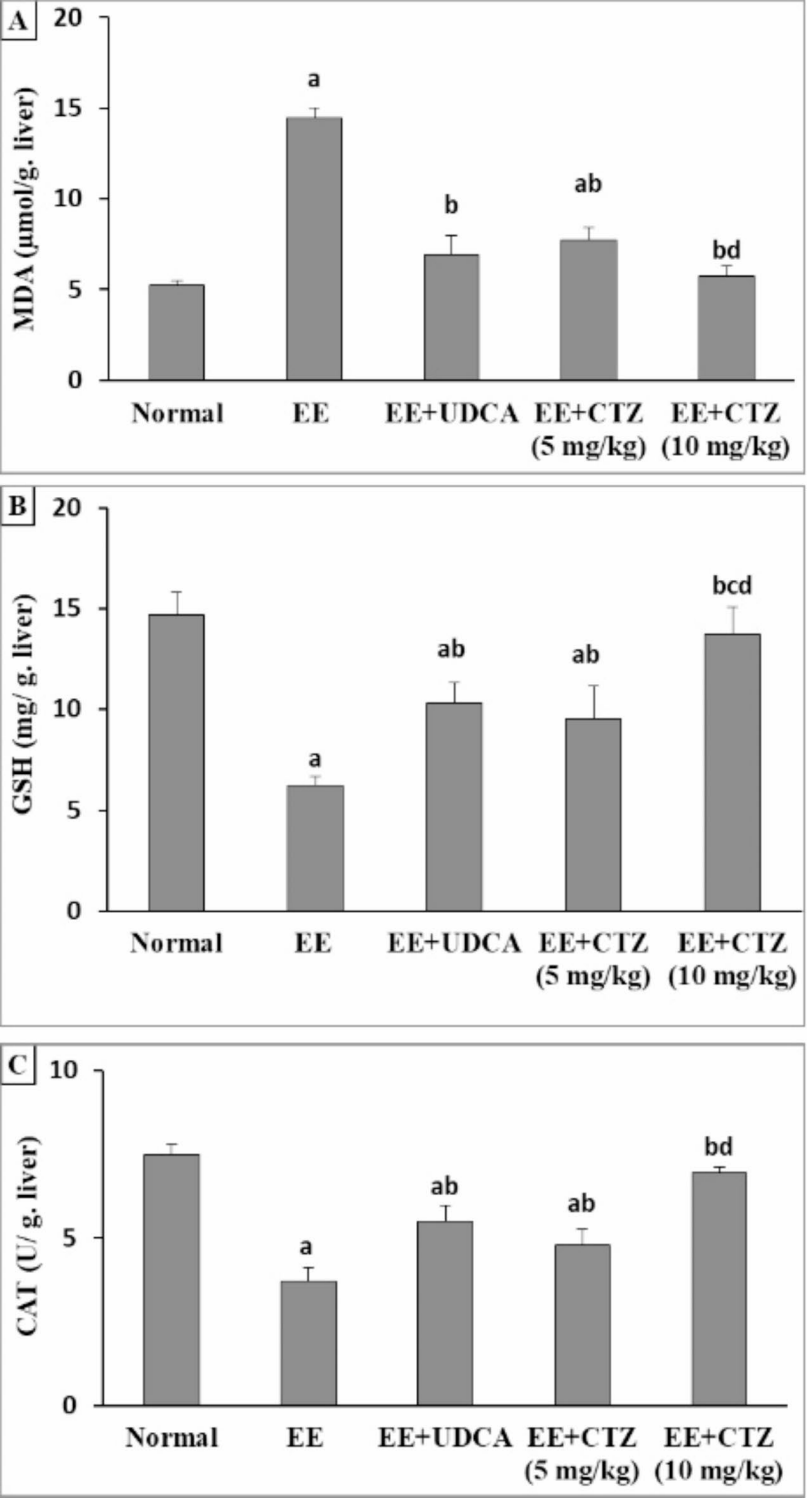
Effect of CTZ on hepatic MDA (**A**), GSH (**B**), and CAT (**C**) contents. *EE* ethinyl estradiol, * UDCA* ursodeoxycholic acid, * CTZ* cilostazol, *MDA* malondialdehyde, *GSH* reduced glutathione, *CAT* catalase. ^a, b, c, d^Significantly different from normal, EE-induced EIC, EE + UDCA, and EE + CTZ (5 mg/kg) groups at *p* < 0.05, respectively.

EE administration resulted in a substantial 2.34- and 2.02-fold decrease in reduced glutathione (GSH) and catalase (CAT) hepatic tissue content, respectively, as well as a considerable rise (2.77-fold increase) in the hepatic malondialdehyde (MDA). In contrast, the administration of UDCA therapy stabilized the hepatic MDA concentration in combination with a drastic increase in the hepatic GSH and CAT contents of 65.02% and 48.65%, respectively, when compared to the EE-intoxicated group. Additionally, treatment with CTZ at a dose of 5 mg/kg resulted in substantial elevation in the hepatic levels of GSH and CAT by 52.72% and 29.19%, respectively. Moreover, there was a notable decline in hepatic lipid peroxidation, as demonstrated by a 46.47% drop in hepatic MDA content. However, at a dosage of 10 mg/kg, CTZ balanced all the oxidative stress markers (Fig. 1).

### CTZ treatment activated HNF1α/FXR pathway

**Fig. 2:**
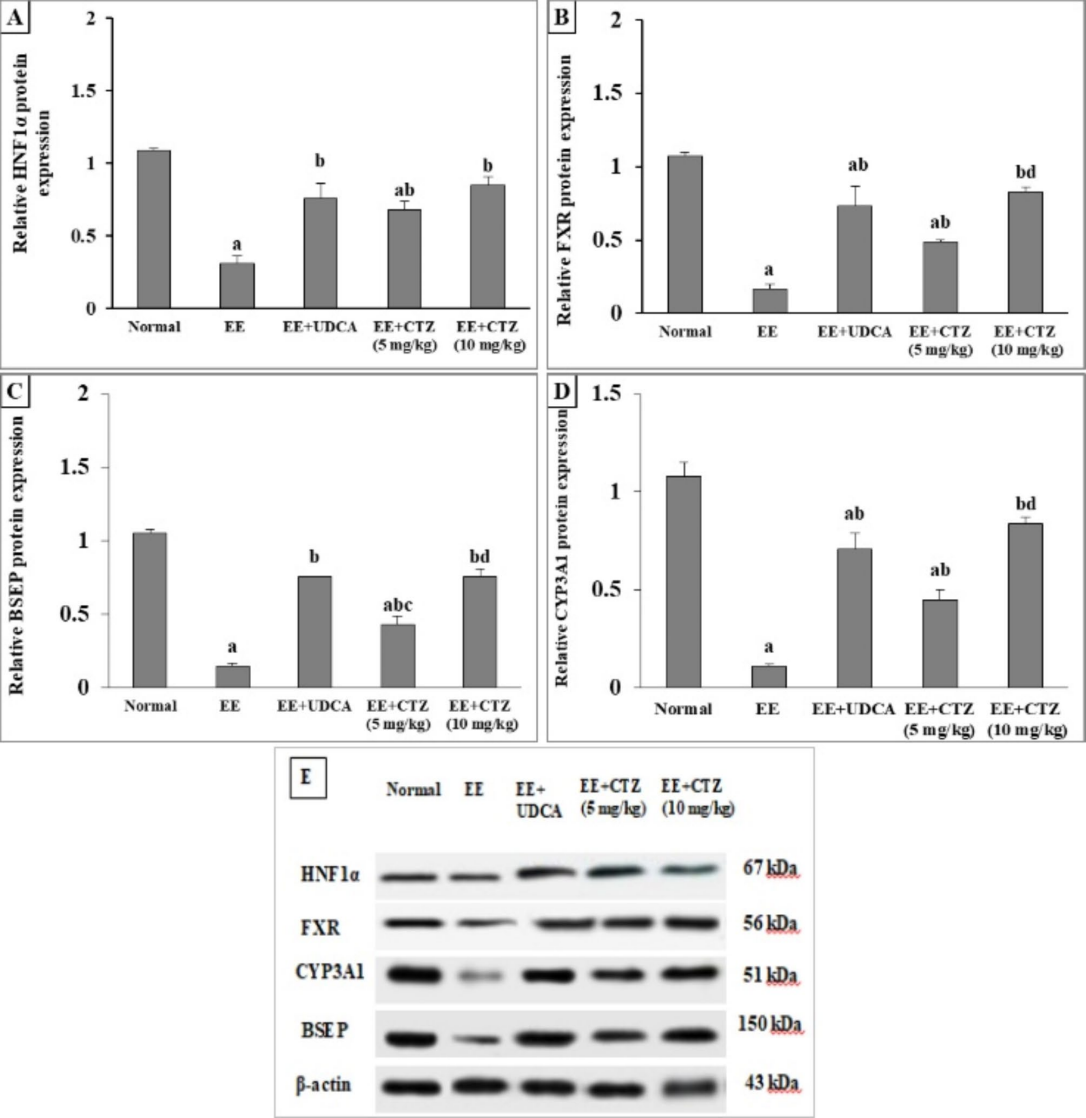
Effect of CTZ on the hepatic protein expression of (**A**) HNF1α, (**B**) FXR, (**C**) BSEP, (**D**) CYP3A1, and (**E**) western blot images. *EE* ethinyl estradiol, *UDCA* ursodeoxycholic acid, *CTZ* cilostazol, *FXR* farnesoid X receptor, *HNF1α* hepatocyte nuclear factor 1α, *CYP* cytochrome P450, *BSEP* bile salt export pump. ^a, b, c, d^Significantly different from normal, EE-induced EIC, EE + UDCA, and EE + CTZ (5 mg/kg) groups at *p* < 0.05, respectively.

Intoxication of rats with EE led to a significant reduction in the relative gene and protein expression of the HNF1α by **2.33** and **3.52**-fold and FXR by **2.65** and **6.53**-fold, respectively, when compared to the normal control group. Similarly, intoxication of rats with EE led to a significant reduction in the relative protein expression of CYP3A1 and BSEP by **9.81 and 7**-fold. Conversely, treatment of these rats with either UDCA or 5 mg/kg CTZ was associated with considerable elevation in the relative gene and protein expression of HNF1α and FXR, along with a significant increment in CYP3A1 and BSEP protein expression (*p* < 0.05). On the other hand, administration of 10 mg/kg CTZ resulted in a more pronounced activation of the HNF1α/FXR pathway, as evidenced by the restoration of their gene and protein expression levels (Fig. 2).

### CTZ treatment modulated retinoid X receptor (RXR), high mobility group box 1 protein (HMGB1), and CYP1A7 gene expressions

The expression of RXR gene was found to be significantly down-regulated in the liver tissues of the group of rats with cholestasis compared to the control group (*p* < 0.05). This reduction was reversed after receiving the UDCA, and 5 mg/kg or 10 mg/kg CTZ (*p* < 0.05) and it was more obvious with increasing the dose of CTZ.

**Fig. 3:**
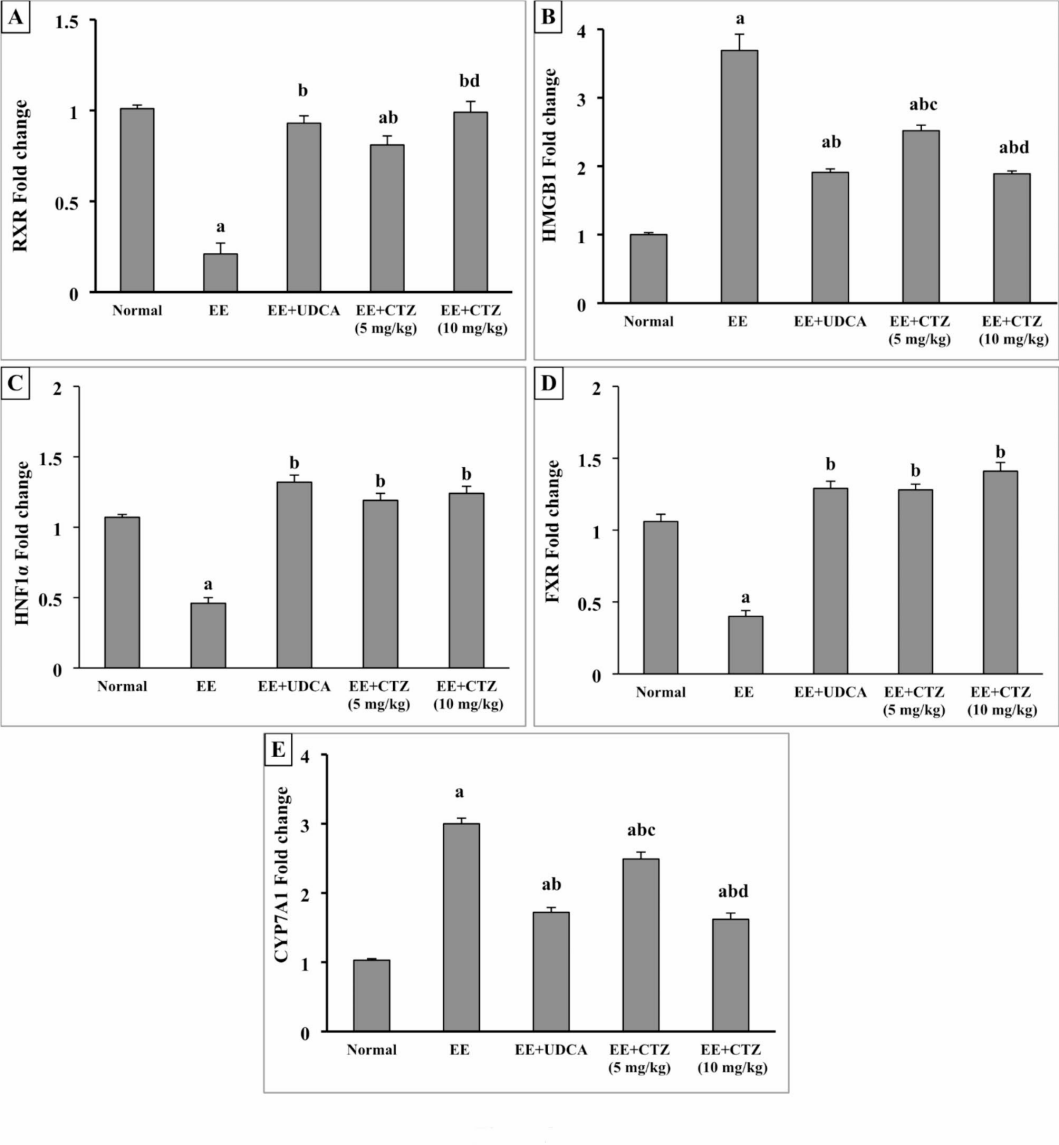
Effect of CTZ on the hepatic gene expression of (**A**) RXR, (**B**) HMGB1, (**C**) HNF1α, (**D**) FXR, and (**E**) CYP1A7. *EE* ethinyl estradiol, *UDCA* ursodeoxycholic acid, *CTZ* cilostazol, *RXR* retinoid X receptor, *HMGB1* high mobility group box 1, *FXR* farnesoid X receptor, * HNF1α* hepatocyte nuclear factor 1α, *CYP1A7* cytochrome1A7. ^a, b, c, d^Significantly different from normal, EE-induced EIC, EE + UDCA, and EE + CTZ (5 mg/kg) groups at *p* < 0.05, respectively.

On the contrary, the CYP1A7 and HMGB1 genes showed a considerable elevation in the EE-injected rats in comparison to the normal rats (*p* < 0.05). Whereas, the administration of UDCA or either dose of CTZ dramatically impeded this up-regulation (*p* < 0.05). However, the high dose of CTZ was more effective than the low dose in down-regulating the expression of CYP1A7 and HMGB1 genes (*p* < 0.05) (Fig. 3).

### CTZ treatment improved liver morphology and histopathological changes induced by EE

**Fig. 4:**
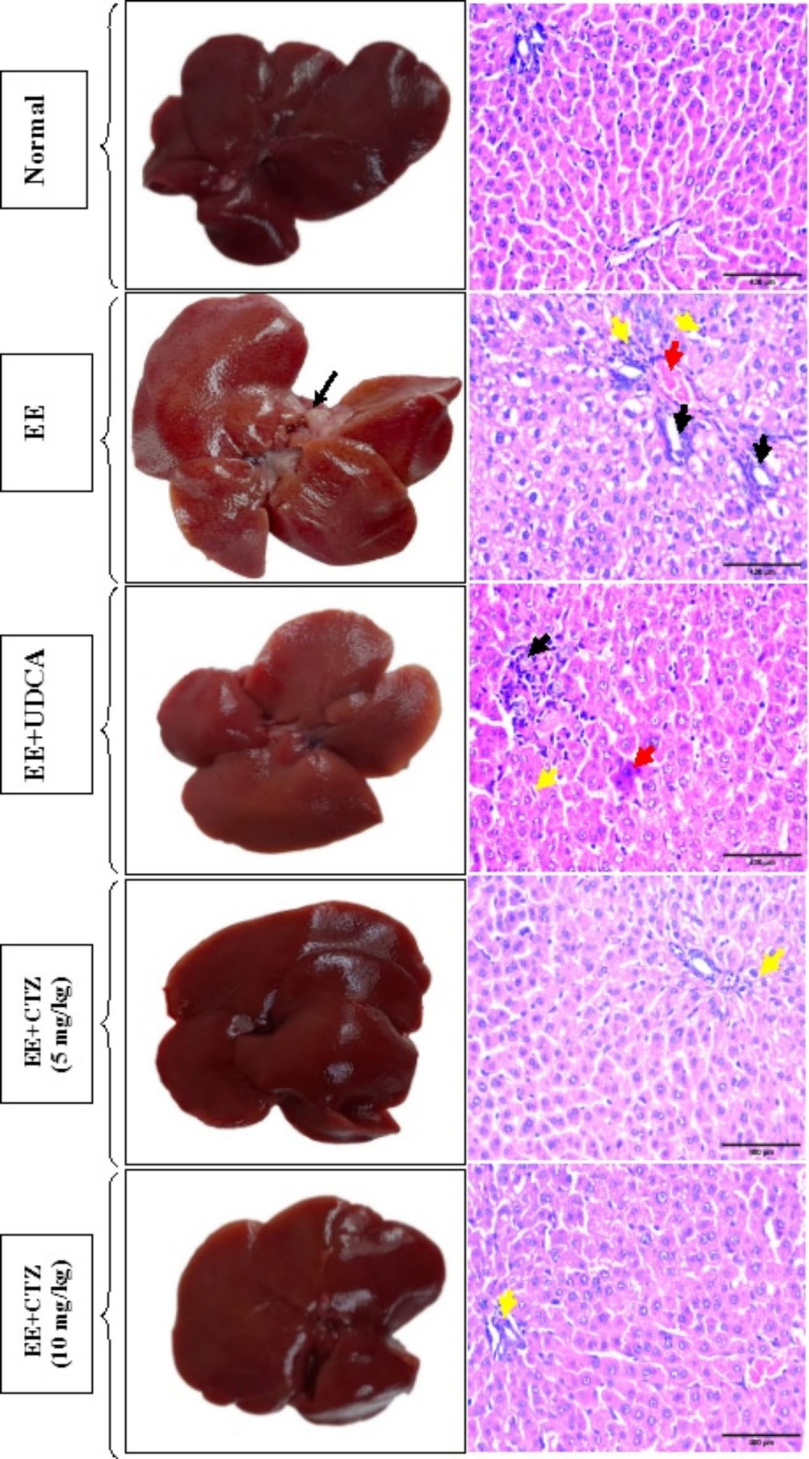
Effect of CTZ on the liver gross morphology and hepatic histopathology (H & E; x400). Red arrows indicate marked congestion, black arrows indicate ductular reaction, and yellow arrows indicate portal and sinusoidal inflammatory cells infiltration. *EE* ethinyl estradiol, *UDCA* ursodeoxycholic acid, *CTZ* cilostazol.

Liver from normal group appeared fresh, slippery and dense both in color and texture. The liver from EE seemed to be enlarged (hepatomegaly) and pale in color with cholestatic plug compared with the normal group. The liver sections obtained from the EIC group exhibited marked congestion, ductular reaction, sinusoidal inflammatory cells infiltration, feathery changes, and portal inflammation with fatty vacuoles. Conversely, hepatic specimens from the UDCA-treated group showed mild hyperemia, mild bile duct proliferation, and scanty portal inflammation. Also, the hepatic tissues from EIC rats treated with 5 mg/kg CTZ revealed almost normal liver lobules with hepatocytes arranged in thin plates, and normal sinusoids. Whereas, in the cholestatic rats given 10 mg/kg CTZ, the hepatic architecture showed normal liver lobule with almost normal hepatocytes organized in thin plates, and normal sinusoids. Furthermore, the liver appearance was improved dramatically, especially in the cholestatic rats given 10 mg/kg CTZ (Fig. 4).

### CTZ treatment improved immnuohistochemical alterations of caspase-3 and Bcl-2 induced by EE

**Fig. 5:**
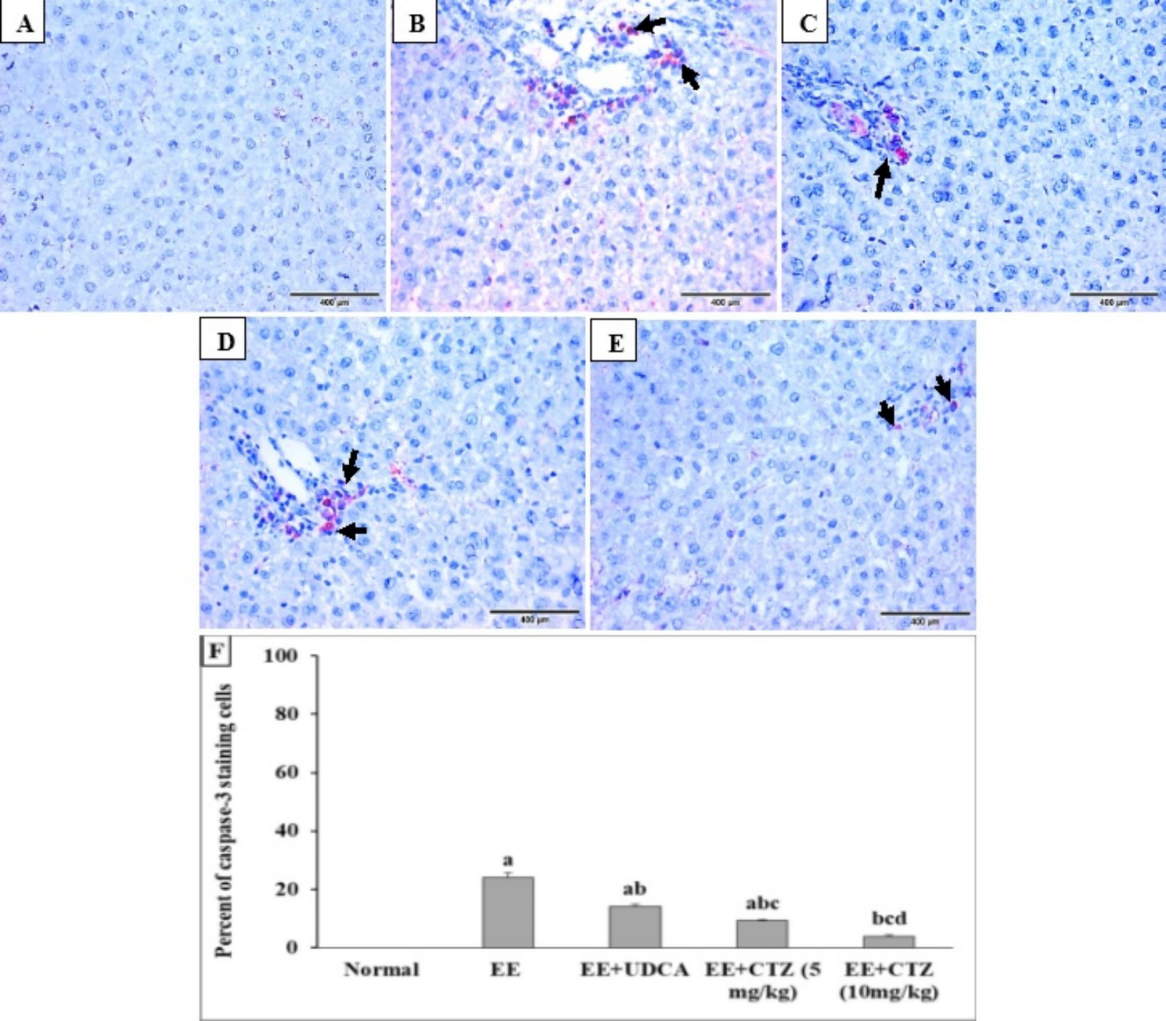
Effect of CTZ on the hepatic caspase-3 immunostaining (DAB, IHC, x400) in normal control (**A**), EE-induced EIC (**B**), EE-induced EIC treated with either (40 mg/kg) UDCA (**C**), a low dose of CTZ (5 mg/kg) (**D**), or a high dose of CTZ (10 mg/kg) (**E**), and (**F**) percentage of caspase-3 immunostaining. *EE* ethinyl estradiol, *UDCA* ursodeoxycholic acid, *CTZ* cilostazol. Black arrows indicate positively stained cells. ^a, b, c, d^Significantly different from normal, EE-induced EIC, EE + UDCA, and EE + CTZ (5 mg/kg) groups at *p* < 0.05, respectively.

Caspase-3 immunostaining was observed to be negative in the liver sections obtained from the normal control group (Fig. 5A). Meanwhile, the hepatic specimens from cholestatic rats had a moderate level of caspase-3 expression, observed as a cytoplasmic brownish stain (24.17%) in the inflammatory cells and lining cells of the bile ducts in the portal system (Fig. 5B). Nevertheless, the UDCA or 5 mg/kg CTZ therapy reduced the expression of caspase-3 (14.17% and 9.33%, respectively) in the inflammatory cells and bile duct lining (Fig. 5C,D,F). Moreover, the administration of a high dosage of CTZ at 10 mg/kg resulted in a further reduction in caspase-3 expression (4%) in the liver (Fig. 5E,F).

**Fig. 6:**
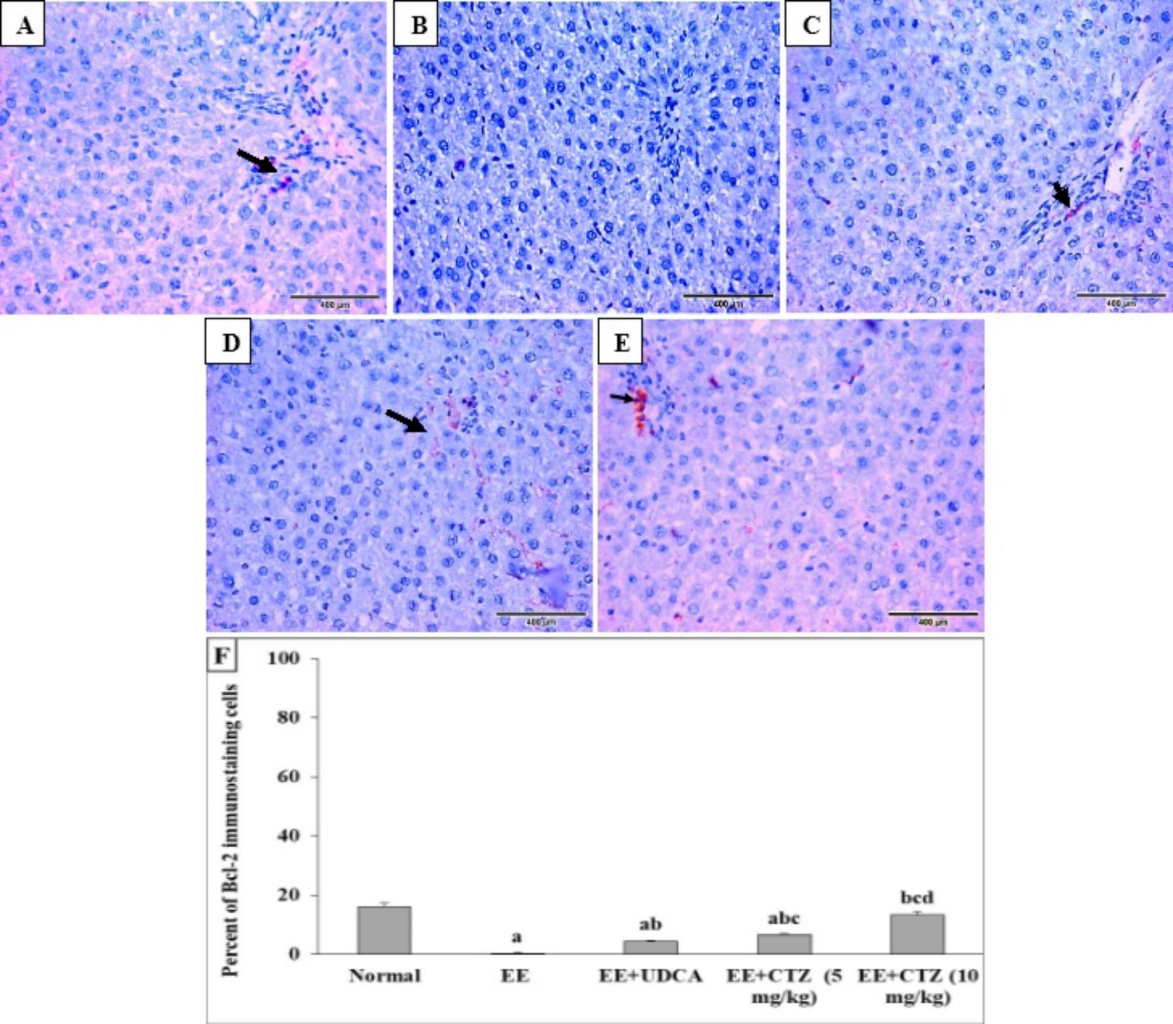
Effect of CTZ on the hepatic Bcl-2 immunostaining (DAB, IHC, x400) in normal control (**A**), EE-induced EIC (**B**), EE-induced EIC treated with either (40 mg/kg) UDCA (**C**), a low dose of CTZ (5 mg/kg) (**D**), or a high dose of CTZ (10 mg/kg) (**E**), and (**F**) percentage of Bcl-2 immunostaining. *EE* ethinyl estradiol, *UDCA* ursodeoxycholic acid, *CTZ* cilostazol, *Bcl-2* B-cell lymphoma 2. Black arrows indicate positively stained cells. ^a, b, c, d^Significantly different from normal, EE-induced EIC, EE + UDCA, and EE + CTZ (5 mg/kg) groups at *p* < 0.05, respectively.

Bcl-2 expression was detected in the inflammatory cells and lining cells of bile ducts in the portal tract (16.17% and 12.67%) in the hepatic tissues from the normal groups of rats, respectively (Fig. 6A,F). This expression was lost (0.33%) upon the injection of rats with EE (Fig. 6B,F). However, liver sections from the animals treated with UDCA or low-dose CTZ exhibited minor positive expression of Bcl-2, which manifested as a cytoplasmic brownish stain (4.50% and 6.67%, respectively) bile duct lining (Fig. 6C,D,F). Whereas, the CTZ, at a dose of 10 mg/kg, nearly restored the normal expression of Bcl-2 (13.50%) in the liver (Fig. 6E,F).

## Discussion

The current study was conducted to elucidate the pharmacological effectiveness of CTZ and the underlying mechanisms via which it exerts its therapeutic benefits in the management of EIC. This was achieved by employing an experimental model generated through the administration of EE. It was revealed that the therapeutic efficacy of UDCA and low dosage of CTZ (5 mg/kg) was comparable. However, when the CTZ was administered at a dose of 10 mg/kg, it resulted in the normalization of all liver function indices, oxidative stress, and pro-inflammatory markers, together with improvement in the histopathological derangements. These effects were mediated through the activation of the HNF1α/FXR pathway.

ALT and AST are recognized as indicators of hepatocyte destruction. In addition, the total bilirubin levels are often utilized to evaluate cholestasis in liver disease patients^18^. In the present study, the levels of ALT, AST, total bilirubin, and ALP were significantly increased in the EE-induced cholestasis group. This increase was substantially mitigated when the EIC rats were given the UDCA or 5 mg/kg CTZ, and it was entirely reversed through the administration of 10 mg/kg CTZ. Also, the UDCA demonstrated an improvement in the hepatic architecture abnormalities caused by the injection of EE, including the marked congestion, ductular reaction, sinusoidal inflammatory cells infiltration, feathery changes, and portal inflammation with fatty vacuoles. This improvement was more pronounced in the EIC rats subjected to a low dosage of CTZ and it led to a complete restoration of the normal hepatic architecture in the animals treated with a high dosage of CTZ. The aforementioned findings reflect the potent ability of CTZ to stabilize the hepatocellular membranes and maintain their structural integrity. Similarly, previous studies have reported the therapeutic benefits of CTZ in the context of cholestatic hepatic injury^19,20^. The findings of these studies indicated that the administration of CTZ caused improvement in hepatic functions, reduction in ductular proliferation, suppression of portal inflammation, decrease in portal hypertension, and amelioration of fibrosis and cholestatic liver injury^19,20^.

Excess estrogen exposure can induce massive oxidative stress and pro-inflammatory cytokine production in the liver^21^. Coinciding with our results, numerous studies have demonstrated that estrogens cause a notable depletion in the hepatic content of GSH and CAT leading to the peroxidation of membrane lipids and the generation of free radicals. These processes subsequently exacerbate the oxidative damage, which is implicated in the initiation and progression of cholestatic liver injury^5,22^. Nevertheless, CTZ treatment resulted in an augmentation of the antioxidant enzyme activities, as indicated by elevated levels of GSH and CAT, while suppressing the production of MDA, an end product of lipid peroxidation. Notably, these effects were more pronounced when higher doses of CTZ were utilized^20^. In the present study, the administration of CTZ at a dose of 10 mg/kg exhibited the ability to effectively restore the balance of all the oxidative stress indicators.

HMGB1 is a powerful pro-inflammatory cytokine that is typically located in the nucleus. HMGB1 undergoes notable posttranslational modifications when cells are activated or undergo cell death. These modifications cause HMGB1 to relocate to the cytoplasm and eventually be released into the extracellular space^23^. In instances of liver tissue injury, such as in cholestasis, HMGB1 is released resulting in inflammation and progression of the disease^24^. In the present study, the cholestatic rats exhibited higher expression levels of the HMGB1 gene, which were subsequently decreased following the administration of UDCA or CTZ, particularly the high dose. Coinciding with these findings, prior research has demonstrated that cholestasis induction is associated with a significant increase in the hepatic expression of HMGB1^25^. Furthermore, there have been reports indicating that the administration of CTZ has the potential to mitigate organ damage and stimulate the expression of heme oxygenase-1 (HO-1), which subsequently contributes to the decrease in circulating HMGB1 levels^26^.

BAs homeostasis is tightly controlled through regulating the BAs synthesis, metabolism, and transport by the hepatic nuclear receptors, including the FXR. One of the mechanisms through which the FXR exerts its action is inhibiting the CYP7A1, hence lowering the BAs production^27^. Also, it has been found that HNF1α is a transcriptional regulator of the FXR and it indirectly down-regulates the expression of CYP7A1 by binding to the FXR receptors^28^. The FXR operates as a heterodimer with another nuclear receptor known as the RXR. This dimerization induces allosteric conformational changes which may enhance the transcriptional activity of FXR and the ability to bind the FXR response elements (FXREs)^29^.

In the current study, the injection of rats with estrogen resulted in down-regulation of the HNF1α, FXR, and RXR with subsequent decreased expression of BA metabolizing enzyme; CYP3A1 and BA transporter; BSEP, as well as enhanced expression of the CYP7A1 gene. Meanwhile, the concurrent administration of the UDCA or CTZ effectively mitigated the effects of estrogen. Notably, the high dose of CTZ demonstrated greater efficacy in alleviating the suppressive effect of estrogen on the HNF1α/FXR pathway and the development of cholestasis.

It has been suggested that the occurrence of inflammation and the accumulation of hydrophobic BAs in cholestasis can ultimately result in the apoptosis of hepatocytes^20,30^. Regarding the proteins related to cell apoptosis, in the current study, the caspase-3 was significantly up-regulated while the Bcl-2 was down-regulated in the EIC rats. These alterations were reversed when the rats were treated with the UDCA or CTZ while the high dose of CTZ restored the normal expression levels of these proteins. In accordance with the results of this study, it was revealed that Bcl-2 expression was dramatically reduced, whereas cleaved caspase-3 was significantly increased in the bile duct-ligated group of rats^31^. Additionally, CTZ significantly depleted the elevated caspase-3, indicating a protective and anti-apoptotic effect of CTZ against thioacetamide-induced liver fibrosis^32^.

UDCA affects the liver through various complex mechanisms, such as altering the bile acid composition, acting as a cytoprotectant, and functioning as both an immunomodulator and a choleretic. It significantly lowers the biliary cholesterol saturation by inhibiting cholesterol absorption in the intestine and reducing its secretion into bile^33^. UDCA’s cytoprotective effects are attributed to its ability to shield hepatocytes and cholangiocytes from bile acid-induced damage, which is mediated by reactive oxygen species that lead to inflammation and mitochondrial dysfunction^34^. It helps cellular integrity, activates anti-apoptotic pathways, and reduces oxidative stress in hepatocytes by inhibiting reactive oxygen species production in Kupffer cells^35^. Therapeutic doses of UDCA convert bile acids from hydrophobic to hydrophilic forms, thereby decreasing the levels of harmful hydrophobic bile acids like deoxycholic and chenodeoxycholic acids, which can damage hepatocytes by increasing membrane permeability and triggering apoptosis^36^. Nevertheless, the advantage of using CTZ over UDCA lies in its lower toxicity. UDCA can have potentially harmful effects, as it is broken down into toxic lithocholic acid after being absorbed in the small intestine. Following hepatic conjugation, UDCA is not further broken down in the liver or intestinal mucosa, leading to the formation of 7-keto-lithocholic acid or lithocholic acid. Lithocholic acid can be toxic to liver cells, potentially causing liver failure in individuals with impaired sulfation, as well as bile duct injury, hepatocyte failure, and cell death^37^. In contrast, CTZ’s toxicity is only observed at high doses. The oral lethal dose in rats and mice is significantly higher, exceeding 5 g/kg. Although data on acute CTZ overdose in humans is limited, potential symptoms include severe headache, diarrhea, hypotension, tachycardia, and possibly cardiac arrhythmia. Due to cilostazol’s high protein binding, dialysis is unlikely to be effective, though gastric lavage may be considered if necessary^38^. The present study provides evidence that the administration of CTZ, in a manner that is contingent upon dosage, has a protective effect against EE-induced cholestatic liver injury. This effect may be attributed to the mitigation of oxidative stress, the reversal of pro-inflammatory cytokines expression, and the regulation of apoptotic cell death. The potential mechanisms underlying these effects may involve the modulation of the HNF1α/FXR pathway and its associated downstream genes. The aforementioned data suggest that CTZ exhibits significant promise as a therapeutic agent for the treatment of cholestatic liver disorders.

## Materials

### Animals

Adult female Sprague-Dawley rats weighing 120–150 g at the start of the experiment were procured from Theodor Bilharz Research Institute’s (TBRI) Schistosome Biology Supply Center (SBSC) in Giza, Egypt. All animals were allowed to acclimatize for 1 week prior to the trials and were kept at a constant temperature (25 °C) and 50% humidity with a 12-h/12-h light/dark cycle; all rats had unlimited access to water and food.

### Drugs and doses

EE (Folone^(R)^; Misr Company for Pharamaceuticals, El-Asher Men Ramadan, Cairo, Egypt, batch number: 19606007) was administered subcutaneously once daily for five days at a dose of 10 mg/kg body weight^39^. UDCA (Ursofalk^(R)^; MINAPHARM, under license from Dr. FALK PHARMA-Germany, batch number: KDE1373) was freshly suspended in 2% cremophore-El (Sigma-Aldrich, St. Louis, MO, USA) before administration via oral gavage at a dose of 40 mg/kg body weight of UDCA for five days^40^. CTZ (Claudol^(R)^; Sabaa International Company for Pharmaceuticals and Chemical Industries, Egypt, Batch number: 19068) was freshly suspended in 2% cremophore-El (Sigma-Aldrich, St. Louis, MO, USA) before administration via oral gavage at a dose of either 5 mg/kg or 10 mg/kg^32^.

### Experimental design

Thirty female Sprague-Dawley rats were split into five groups of six animals each: (I) Normal group, (II) EE-induced intrahepatic cholestasis group, (III) EE + UDCA-treated group, (IV) EE + CTZ (5 mg/kg)-treated group, and (V) EE + CTZ (10 mg/kg)-treated group.

Rats were sacrificed by decapitation under mild anaesthesia (ketamine 87 mg/kg and xylazine 13 mg/kg)^41^ 24 h following the administration of treatment. Blood samples were collected and sera were separated by centrifugation at 1850 xg for 10 min before being frozen at -80 °C for liver function tests. After washing with ice-cold saline, the liver was promptly excised and obtained for assessing oxidative stress markers, extracting RNA, and conducting western blot analysis. Additionally, a portion of the liver was stored in 10% formalin for histo- and immunohistochemical examinations.

### Assessment of liver injury and oxidative stress markers

Serum biomarkers of liver function, such as ALT, AST, and ALP, as well as direct, indirect, and total bilirubin, were quantified using commercially available kits (Biodiagnostics, Egypt) according to the manufacturer’s instructions. To assess oxidative stress markers, liver tissue was homogenized in 10 volumes (w/v) of ice-cold 0.1 M potassium phosphate buffer (pH 7.4) containing 1 mM EDTA, followed by centrifugation for 10 min at 600 xg and then for 20 min at 10,000 xg at 4 °C. The supernatant was collected and stored at -80 °C for analysis of lipid peroxidation, which is indicated by the production of MDA, GSH, and CAT, in liver tissues. This analysis was conducted using commercially available kits (Biodiagnostics, Egypt) as directed by the manufacturer.

### Gene expression analysis of RXR, CYP1A7, and high mobility group box 1 (HMGB1)

Total RNA was extracted from liver samples and reverse transcribed using QIAamp RNA Mini Kit and QuantiTect Reverse Transcription Kit (Qiagen, USA), respectively, following the manufacturers’ protocol. Gene expression was relatively quantified by real-time PCR using TaqMan gene expression master mix (Invitrogen, Germany) according to the manufacturer’s instructions. All the primers used for the amplification of the HNF1α, FXR, RXR, HMGB1, and CYP1A7 genes were ready-made primers purchased from Thermoscientific, USA. The relative quantities of the target genes were calculated against a reference gene, β-actin. The relative expression was determined using the comparative cycle threshold (Ct) (2^−ΔΔCT^) method.

### Western blot analysis of HNF1α, FXR, CYP3A1, and BSEP

The Ready PrepTM total protein extraction kit (Bio-Rad Inc; CAT no: 163–2086) was used to separate proteins from stomach homogenates. For quantitative protein analysis, the Bradford Protein Assay (Markham, Ontario, L3R 8T4, Canada; CAT no: SK3041) was given. Tissue samples (20 g) were loaded with an equal volume of 2x Laemmli sample buffer containing 4% sulfate polyacrylamide gel (SDS), 10% 2-mercaptoethanol, 20% glycerol, 0.004% bromophenol blue, and 0.125 M Tris HCl before being transferred into PVDF membranes. For 2 h at room temperature, the membranes were blocked in tris-buffered saline with 0.1% Tween-20 (TBST) and 5% nonfat milk. The membranes were incubated with primary antibodies against HNF1α (Abcam, USA; CAT no: ab128912), FXR (Santa Cruz, USA; CAT no: sc-25309), CYP3A1 (Santa Cruz, USA; CAT no: sc-53246), BSEP (Proteinintech, USA; CAT no: 67512-1-lg), and β-actin (dilution 1:1000) overnight at 4 °C. Then, they were rinsed with TSBT and incubated with HRP-conjugated secondary antibody (Goat anti-rabbit IgG-HRP-1 mg Goat mab-Novus Biologicals) solution against the blotted target protein. The chemiluminescent substrate was then added to the blot (ClarityTM Western ECL substrate Bio-Rad; CAT no:170–5060). On the ChemiDoc MP imager, the chemiluminescent signals were collected using a CCD camera-based imager, and image analysis software was used to read the band strength of the target proteins against the control sample β-actin by protein normalization.

### Histopathology examinations

Paraffin blocks containing liver tissues that had been stored in a 10% formalin solution were made. The embedded wax blocks were split into 4 m thick sections. Following xylene dewaxing, the slides were stained with hematoxylin and agitated for 30 s before being rinsed in H_2_O for 1 min and stained with 1% eosin solution for 30 s with agitation, all at room temperature (20–25 °C). Five paraffin liver slices were made for each rat to study histopathological alterations (magnification x400).

### Immunohistochemical evaluations of caspase-3 and Bcl-2

Immunohistochemistry for caspase-3 and Bcl-2 was performed on paraffin-embedded tissue slices of 5 m thickness. Sections were pre-treated with the proteolytic enzyme proteinase K (Agilent Dako, CA, USA) and then washed in phosphate-buffered saline for 5 min to disclose the antigens. After that, slices were incubated for 60 min at 37 °C with a primary antibody against caspase-3 and Bcl-2 (Santa Cruz Biotechnology, USA). After washing with, a secondary antibody (Agilent Dako, CA, USA) was applied for 60 min. The reaction was visualised using 3,3’-diaminobenzidine (DAB) chromogen (Agilent Dako, CA, USA). For each rat, the percentages of caspase-3 and Bcl-2 positively stained brown cytoplasmic staining in 10 consecutive fields (magnification x400) were determined.

### Statistical analysis

The data were presented in the form of mean ± SEM. The statistical software package SPSS, version 16.0 (Chicago, IL, USA), was employed to conduct a one-way analysis of variance (ANOVA) test, followed by a Tukey’s test. This analysis aimed to evaluate the presence of statistically significant differences among the mean values of the different groups. Results were deemed statistically significant when the *p-*value was less than 0.05.

## Electronic supplementary material

Below is the link to the electronic supplementary material.

Supplementary Material 1

## Data Availability

The datasets used and/or analyzed during the current study available from the corresponding author on reasonable request.
