# Supplementary material for: Protective effects of cilostazol via the HNF1α/FXR signalling pathway and anti-apoptotic mechanisms in a rat model of estrogen-induced intrahepatic cholestasis
Source: Sci Rep. 2024 Oct 1;14:22751. doi: 10.1038/s41598-024-72729-w (PMC11443125; doi:10.1038/s41598-024-72729-w)
Supplement: Supplementary file 1 — Supplementary Material 1 [file 41598_2024_72729_MOESM1_ESM.docx]

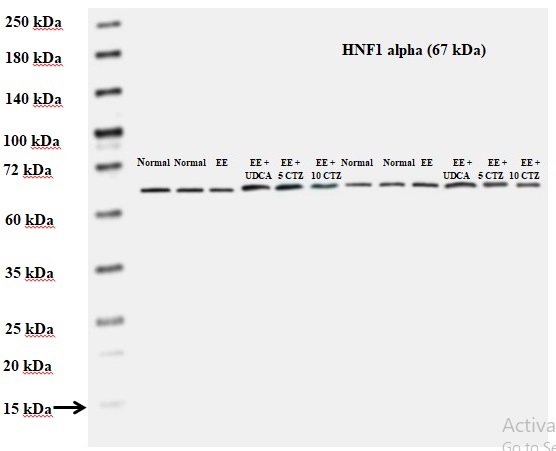


**Fig. S1:** Uncropped gel image from western blot for HNF1α protein


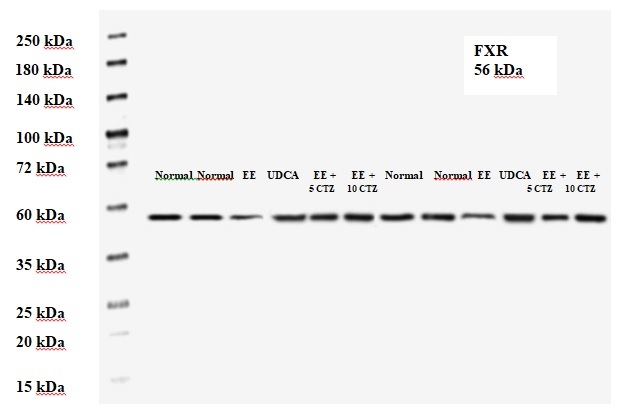


**Fig S2:** Uncropped gel image from western blot for FXR protein

**Fig S3:** Uncropped gel image from western blot for CYP3A1

**Fig S4:** Uncropped gel image from western blot for BSEP protein


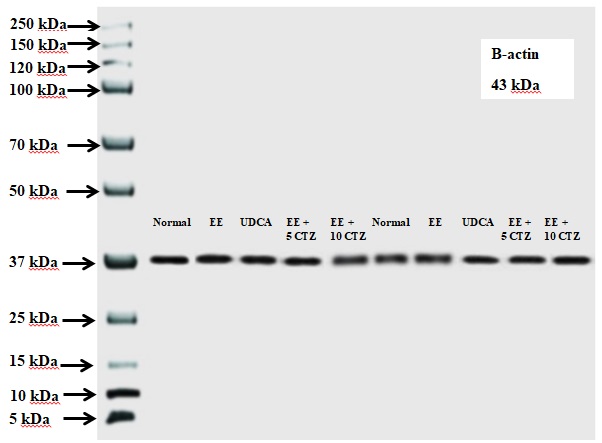


**Fig S5:** Uncropped gel image from western blot for β-actin protein
